# Dual-tasking interference is exacerbated outdoors: A pilot study

**DOI:** 10.3389/fspor.2023.1077362

**Published:** 2023-02-20

**Authors:** Rania Almajid, Kwadwo O. Appiah-Kubi, Daniel Cipriani, Rahul Goel

**Affiliations:** ^1^ Department of Physical Therapy, Stockton University, Galloway, United States; ^2^ Department of Physical Therapy, West Coast University, Los Angeles, CA, United States; ^3^ Department of Physical Therapy, Clarkson University, Potsdam, NY, United States; ^4^ Department of Neuroscience, Baylor College of Medicine, Houston, TX, United States

**Keywords:** multitasking behavior, environment, texting, dual-tasking, posture

## Abstract

**Introduction:**

Walking while texting can create gait disturbances that may increase fall risk, especially in outdoors environment. To date, no study has quantified the effect of texting on motor behavior using different dynamic tasks in outdoor environments. We aimed to explore the impact of texting on dynamic tasks in indoor and outdoor environments.

**Methods:**

Twenty participants (age 38.3 ± 12.5 years, 12 F) had a Delsys inertial sensor fixed on their back and completed walk, turn, sit-to-stand, and stand-to-sit subtasks with and without texting in both indoor and outdoor environments.

**Results:**

While there was no difference in texting accuracy (*p* = 0.3), there was a higher dual-tasking cost in walking time with texting outdoors than indoors (*p* = 0.008).

**Discussion:**

Dual tasking has a greater impact on walking time outdoors compared to an indoor environment. Our findings highlight the importance of patient education concerning dual-tasking and pedestrian safety in clinical settings.

## Introduction

Smartphone use is now a common daily activity for millions of people. In the United States, smartphone use grew from 35% in 2011 to 81% in 2019 (1). The digital expansion has increased the number of annual text message exchanges to 2.1 trillion (2), as well as concerns for pedestrian safety. Using a smartphone can increase the risk of an accident while crossing streets, in virtual and real pedestrian environments: especially among younger adults, who text more often than other age groups (3, 4). These statistics have led to the a growing interest in examining the effect of Texting While Walking (TeWW) (5–7).

Texting is an ecologically valid task that can impact motor behavior and requires fine and gross motor skills control, visual attention, and cognition. Texting ecological validity can be influenced by many factors, such as the text message length, the text's emotional content, memory utilization, environmental factors, and the use of the personal mobile device (8). Previous research showed that adults walk slower with a shorter stride length, a longer stride time, and greater gait variability while texting (5–7). These findings were based on using one mobile device across the participants, which may decrease its application to real-life scenarios. The gait deviations associated with texting may compromise safety and contribute to the rising number of mobile phone-use injuries (9). Although straight walking makes up the majority of steps taken during daily activities (10), we regularly perform many functional activities while interacting within our environments, e.g., standing up from a bench to catch a bus or turning to avoid a hole on the street. In fact, turning comprises up to 45% of the steps that we take daily (10) and can be more challenging than walking (11) as it requires pre-planning the motor path, changing body orientation, and relying on visual guidance (12). Hence, in addition to walking, it is essential to understand if and how texting affects the dynamic tasks that we perform in everyday life.

Movement is constrained by factors related to the individual, task, and environment (13). For instance, the complexity of a motor behavior increases in open unpredictable environments, e.g., outdoor spaces with visual and auditory distractions, compared to predictable closed environments, e.g., indoor quiet spaces (14). This occurs due to the constant need for attention and adaptation to a changing environment. Subsequently, individuals need to modify their motor strategies to maintain postural stability (14). Much of our understanding of dual-tasking interference originates from paradigms based on predictable indoor-laboratory findings with minimal distractions and variability. These indoor-lab settings demonstrate little resemblance to the outdoor, i.e., real-world environments where most falls occur (15). Due to their multifactorial nature, up to 78% of falls of individuals between the ages of 18 and 64 occur outdoors (16). Yet, compared to falls in older adults, falls in younger and middle-aged adults are not fully understood. Outdoor spaces can contain more distractions as individuals attempt to multitask while navigating unpredictable surfaces, noises, moving scenes, and weather changes. These conditions decrease pedestrian safety and increased the likelihood of accidents (3). Thus, understanding the used motor strategies in outdoor environments can advance our knowledge about pedestrian safety and accidents in young and middle-aged adults.

In addition to environmental adaptation, dual-tasking interference leads to decreased gait speed, cadence, stride length, and increased stride time (17); which all depends on the type of postural and secondary tasks being performed. Greater interference has been observed with walking and turning compared to sit-to-stand and stand-to-sit, and with more complex secondary tasks, e.g., texting is more challenging than holding a cup of water (18). Compared to walking in a quiet indoor hallway, adults exhibited greater gait variability and decreased texting accuracy when walking on a flat outdoor sidewalk (5). However, the influence of texting on dynamic tasks in an outdoor environment is largely unknown and has not been quantified. This lack of knowledge is concerning, and it should be an essential focus for research since pedestrian safety concerns are higher among younger adults compared to older adults. These individuals are more likely to engage in risk-taking behaviors, e.g., texting while walking, in unpredictable outdoor environments.

Therefore, we aimed to investigate the effects of texting on four dynamic tasks–walk, turn, sit-to-stand, and stand-to-sit–within indoor and outdoor environments. We used the Timed Up and Go (TUG) test because it requires the completion of serial subtasks: sit-to-stand, turn, walk, and stand-to-sit. We hypothesized that the outdoor environment would lead to greater dual-tasking interference than indoors.

## Materials and methods

### Participants

Twenty participants (age 38.3 ± 12.5 years) gave written informed consent to participate in this study and were recruited using word-of-mouth or flyers. Participants did not receive any compensation for participating in the study. The data of this study are derived from a larger study, part of which has already been published (19), where we assessed the feasibility of an iPhone application in identifying gait changes in dual-tasking conditions. The Institutional Review Board approved the protocol at West Coast University (approval #23574). We confirm that we have reported all the measures, conditions, data exclusion, and the sample size determination.

Inclusion criteria included the ability to walk independently with or without an assistive device and the current usage of a touchscreen smartphone for text messaging. Participants were excluded from the study if they presented with any balance instabilities, cognitive deficits, concurrent musculoskeletal injuries, or neurological conditions that would affect balance.

### Assessment protocol

The participants underwent a performance-based balance and functional mobility battery. The Activity-specific Balance Confidence (ABC) scale is a self-reported questionnaire that allows users to rate their perceived confidence related to balance in 10 different activities of daily living (20). The Berg Balance Scale (BBS) is a widely accepted performance-based balance measure that rates performance on a series of 14 different tasks that include sitting, standing, reaching, turning, looking over each shoulder, standing on one leg, and stepping (20). The Montreal Cognitive Assessment (MoCA) is used to assess cognitive function. For ABC and BBS, we used cut-off scores of <67 and ≤40, respectively, to ensure normal balance function (20). For MoCA, we used a cut-off score of >26 to ensure normal cognitive function (21). The absence of musculoskeletal and neurological conditions was confirmed by a written response from each participant prior to data collection using a questionnaire. Additionally, the questionnaire included questions related to the use of texting/day as a percentage, educational level, height, and weight. One participant was disqualified due to a recent concussion unrelated to this study. The assessment protocol took approximately 20–30 min to complete.

### Experimental protocol

**Figure 1:**
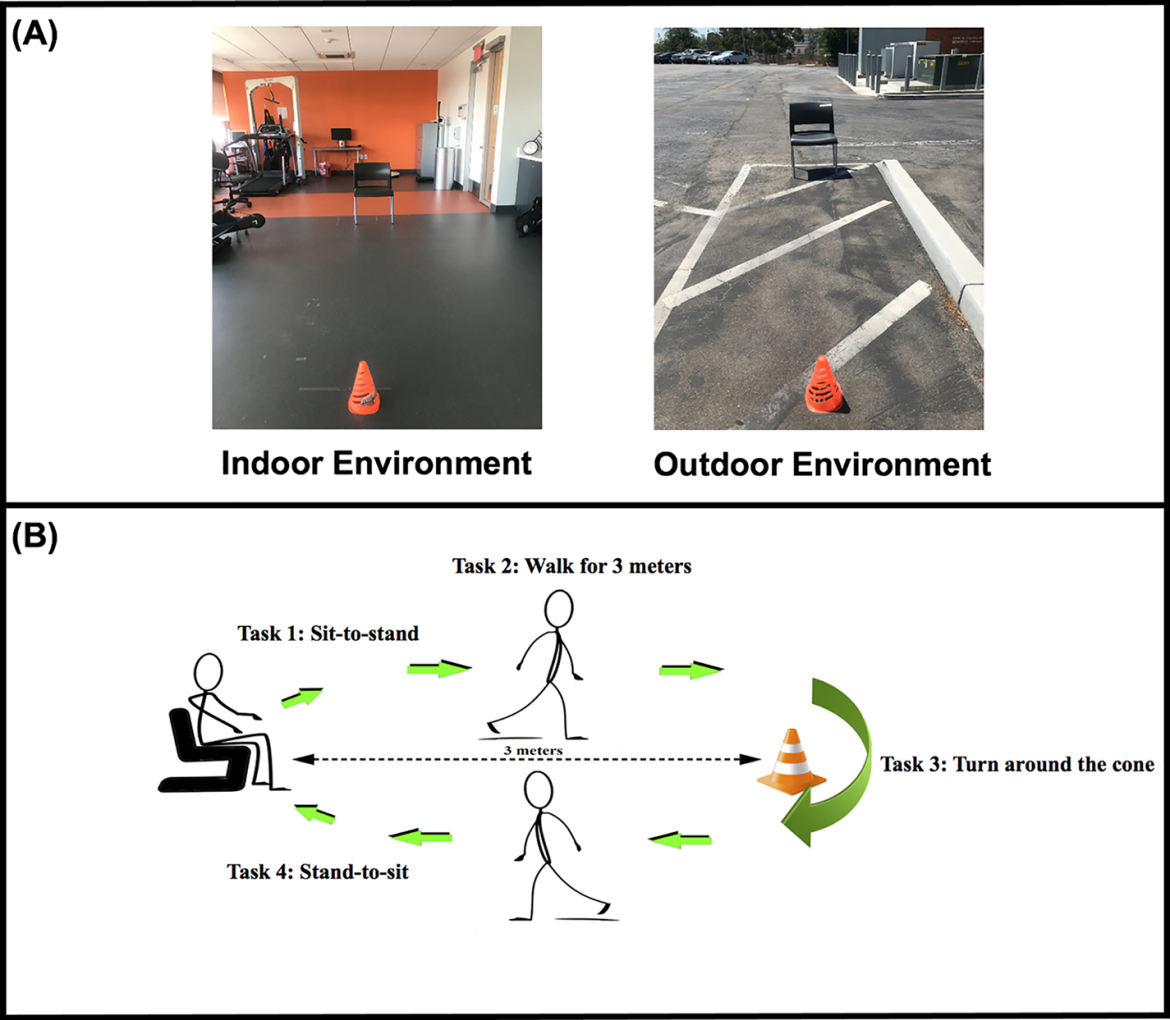
(**A**) The indoor laboratory and outdoor environments experimental setting and (**B**) The timed up and go test and its four subtasks (sit-to-stand, walk, turn, and stand-to-sit).

Participants were assessed in two environments: (1) indoors in a well-lit laboratory room, and (2) outdoors on a sidewalk in the parking lot of a university campus (Figure 1A). The indoor environment was more predictable with no auditory distractions or movements of the people/objects. The outdoor environment contained pedestrians including students and staff entering/leaving the campus, and occasionally, vehicles driving around the parking lot. It consistently had traffic noise and car horns, which created an auditory distraction that could impact an individual's attention and postural responses (22). To measure the auditory distraction at both environments, we used a Decibel Sound Level Meter that provides a measurement with a range = 30–130 dBA, accuracy = ±1.5 dB, frequency response, 31.5 Hz–8 Khz, and resolution = 0.1 dB (XRClif Shenzhen XRC Electronics Co., LtD), however, the auditory distraction was not compared between conditions. The indoor sound average was 41.2 dB [standard deviation (SD) = 3.64 dB], with a minimum of 37.3 dB and a maximum at 47 dB, whereas the outdoor sound average was 84.3 dB (SD = 9.2 dB), with a minimum of 67.3 dB and a maximum 110.2 dB. All the participants were tested in Los Angeles, CA between the dates June 5th and September 6th and each testing session was conducted in sunny weather with temperatures ranging from 61 to 88°F. The participants were asked to complete two experimental conditions in both settings: (1) TUG without texting and (2) TUG with texting. The order of the environment was counterbalanced; half of the participants were assigned to begin the protocol in an indoor environment, and the other half started outdoors. The order of the conditions within each environment (single-indoor and dual-indoor or single-outdoor and dual-outdoor) was randomized before testing using Excel software (Microsoft, Redmond, WA, United States). When performing the TUG simultaneously with texting, participants were instructed to focus on both (i.e., walking and texting tasks); if this was not possible, they were instructed to focus on moving safely. We refrained from using prioritization instructions to generate a more ecologically valid task. Participants were asked to perform the TUG protocol at their normal walking speed. The two experimental conditions were:

(1) **TUG:** participants were given verbal instructions to stand up from an armless chair (back height: 80 cm, seat height: 39.4 cm, width: 45.7 cm, length: 50.8 cm); when the examiner said “go,” they walked to an orange cone placed 3 meters in front of them, turned around the cone, walked back, and turned to sit on the chair (Figure 1B).
(2) **TUG while texting:** participants were asked to perform the TUG while texting a response on their smartphone to a verbal question. In the indoor environment, all participants were asked, “What did you eat for breakfast this morning?” and in the outdoor environment, they were asked, “What are you doing this evening?” Participants were instructed to make an attempt to type full-sentence responses. Text assistants such as auto-correction and auto-capitalization were turned off before these experiments.

To determine step counts in the TUG, each task was videotaped with a camera (SONY Cyber-Shot DSC-W800, San Diego, CA, United States) fixed on a tripod and placed approximately 2 meters away from the cone (i.e., where the participants turned). A Trigno™ Avanti wireless Inertial Measurement Unit (IMU) sensor with a sampling frequency of 148 Hz (Delsys Inc., Natick, MA, United States) was placed on the participants' lumbar spine (L2 level) using Mueller wrapping (23, 24) to detect and measure the sit-to-stand, stand-to-sit and, and turn during the TUG. To note, each participant also had a smartphone that was fixed at the waist level using a belt. The smartphone data were used to answer another research question, and is presented elsewhere before (19).

### Data analysis

**Figure 2:**
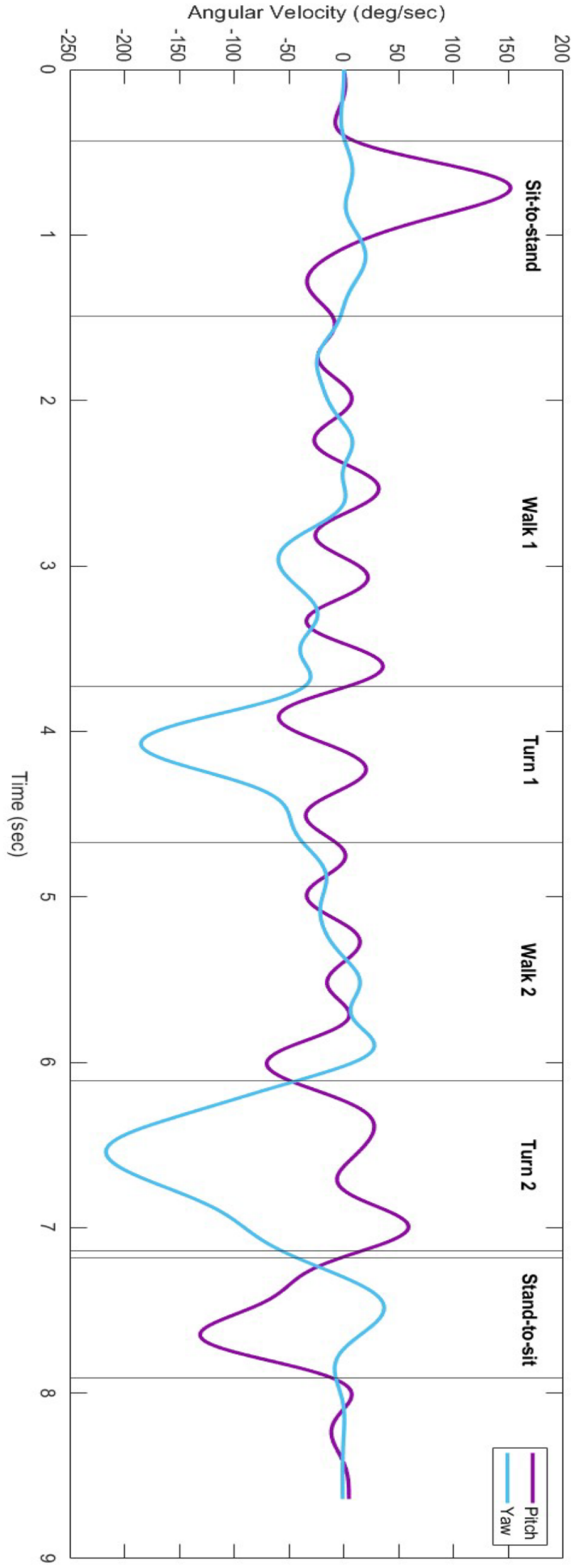
An example of a filtered signal taken from the lumbar sensor showing the pitch (purple) and yaw (blue) angular velocity signals (deg/sec) from a participant while completing the TUG test.

We analyzed all the signals from the IMU sensor using custom MATLAB scripts (MathWorks, Natick, MA, United States). To decrease the noise of the signals, the gyroscope signals were filtered using a fourth-order Butterworth low-pass filter with a cut-off frequency of 2 Hz (25). Consistent with previous studies (26, 27), we used the first 10°/s and the second −10°/s of the pitch angular velocity signal to determine the start and end of the sit-to-stand, respectively. Then, we used the last 10°/s and the last −10°/s of the pitch angular velocity signal to mark the start and end of stand-to-sit, respectively. To detect the turning tasks, i.e., turn 1 and turn 2, we used the yaw angular velocity signal when it exceeded (start of turn) and diminished (end of turn) >20% of each participant's maximum yaw angular velocity. The walking tasks then were demarcated as follows: (a) walk 1 (from chair to cone): starts from the end of sit-to-stand and ends at the start of turn 1, and (b) walk 2: starts from the end of turn 1 and ends at the start of turn 2 (Figure 2).

The dependent variables were as follows:

(1) TUG subtask times (sec): the times to complete the TUG subtasks were calculated using the IMU sensor (23). The TUG is described above, and its subtasks included sit-to-stand, walk, turn, and stand-to-sit as shown in (Figure 1B). The walking time was average for walk 1 and walk 2 and the turning time was averaged for turn 1 and turn 2.
(2) Gait speed (m/sec): this variable was calculated as the average of walk 1 and walk 2 using the IMU sensor, defined as the time it takes the participant to travel the entire distance. The gait speed is sensitive to dual-tasking (17).
(3) Step count (step): number of steps taken by the individual to complete the TUG; it is sensitive to dual-tasking (28).
(4) The dual-task costs (DTCs) of the TUG subtask times, step count, and gait speed: the relative change in these variables in dual-tasking conditions compared to single-task conditions. DTC is a measure of cognitive-motor interference (29) and is demonstrated as a % calculated with the following equation for all the variables except gait speed:

$$\mathrm{DTC}\;=\;-\frac{\left[\left(\mathrm{Dual}\;\mathrm{task}\;-\mathrm{Single}\;\;\mathrm{task}\right)\right]}{\left(\mathrm{Single}\;\mathrm{task}\right)}\times \;100.$$

Higher gait speeds indicate better performance; thus, we used the same DTC formula without a negative sign as follows:

$$\mathrm{DTC}\;=\frac{\;\left[\left(\mathrm{Dual}\;\mathrm{task}\;-\mathrm{Single}\;\mathrm{task}\right)\right]}{\left(\mathrm{Single}\;\mathrm{task}\right)}\times \;100.$$

A negative DTC indicates a poorer performance or an increase in DTC (28).

(5) Number of responses and accuracy (%) in texting task: Number of responses was calculated by counting the correct and incorrect responses in texting task. Accuracy was evaluated by counting the number of correct and incorrect responses in the texting tasks. Incorrect responses were defined as spelling errors, unwanted, or missing words. This was calculated as:

$$\mathrm{Accuracy}\;=\;\frac{\mathrm{Number}\;\mathrm{of}\;\mathrm{correct}\;\mathrm{responses}}{\mathrm{Total}\;\mathrm{responses}\;}\;\times \;100.$$

### Statistical analysis

The study was powered to detect a large effect size since small to medium effect sizes would not be practical for healthy adults (6). We calculated the sample size based on an analysis conducted by G*power 3.1 (University Kiel, Germany) (30). A minimum sample size of 20 was required to achieve a statistical power of 0.8 for an effect size of 0.6 at an alpha level of 0.05 (6) for the Wilcoxon Signed-Rank test. The Wilcoxon Signed-Rank test used to detect the difference between the dependent variables, in addition to the difference in response rate and accuracy while texting in indoor and outdoor environments. As a result, a sample size of 20 was deemed sufficient to test the study hypothesis. The Shapiro-Wilk test was performed before the analyses to assess the normality assumption of the distribution for the dependent variables. Between-condition (indoors vs. outdoors) differences were assessed using the Wilcoxon Signed-Rank test for non-normally distributed variables and paired *t*-test for normally distributed variables. We calculated the effect sizes (ES) using Rosenthal's equation *r* = Z or T score/√N (31), where the N is the sample size, and Z or T are determined by Wilcoxon Signed-Rank tests or paired *t*-tests, respectively. Values of 0.1, 0.3, 0.5, and 0.7 represent small, medium, large, and very large effect sizes, respectively (32). All statistical analyses were conducted using the SPSS 26 package (IBM, Armonk, NY, United States) with an alpha level of 0.05.

## Results

### Demographic and clinical characteristics

Participant characteristics and results are presented in Table 1. The mean and SD for participants' age was 38.3 ± 12.5 years; with 60% of them being female. The mean score and SD of the ABC was 98.3 ± 1.8, BBS was 55.5 ± 1.8, and MoCA was 28.7 ± 1.1, reflecting normal cognition and balance. Participants reported that they used their mobile phones to browse the internet and text more than conducting phone calls.

**Table 1:** Demographics and other basic assessment measures.

Characteristics	(*n* = 20)		
	Mean	SD	Range
Age (years)	38.3	12.5	25–62
Gender	Male: 40%, Female: 60%	—	—
BMI (kg/m^2^)	27.3	5.9	21.8–45.2
Education Level			
Undergraduate (%)	50%		
Graduate (%)	50%		
Hand Dominance			
Right	90%		
Left	10%		
Time with Current smartphone (months)	21.4	12.5	1–48
Use of Smartphone (%)			
Calls	14.6	10.6	3–40
Texts	30.1	13.1	5–50
Internet	55.2	19.4	20–90
BBS (0–56)	55.5	1.8	48–56
ABC (0–100)	98.3	1.8	93–100
MoCA (0–30)	28.7	1.1	27–30

Values of mean, SD, and range are represented. BMI, body mass index; BBS, berg balance scale; ABC, activity-specific balance confidence scale; MoCA, the Montreal cognitive assessment.

### Effect of environment

**Figure 3:**
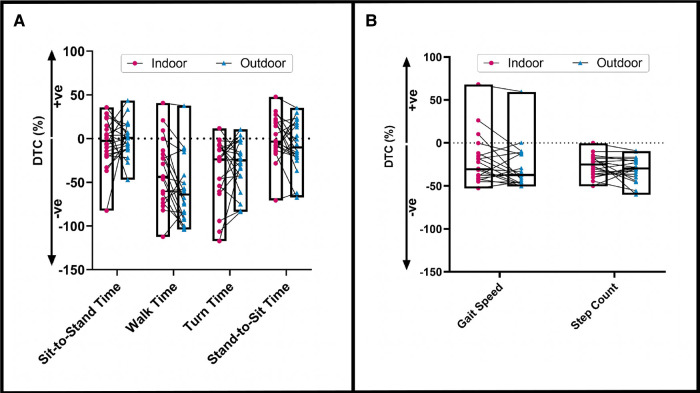
The dual-tasking cost (DTC) of the (**A**) sit-to-stand time, walk time, turn time, and stand-to-sit time and (**B**) gait speed and step count in indoor (in red color) and outdoor (in blue color) environments while texting. The box plot depicts the median values of DTCs. To note, (+ve) indicates an improvement in dual-tasking performance, and (−ve) indicates a decline in dual-tasking performance.

The dependent variables and their DTCs are presented in Table 2. Participants took a longer time to walk outdoors than indoors (*p *= 0.01). In the outdoor environment, walking time DTC was significantly larger than indoors, reflecting a poorer performance when the participants completed the texting (*p *= 0.008) task. A non-significant trend emerged in our data, suggesting that participants had higher turning time DTCs in the outdoor environment while texting (*p *= 0.057). No significant differences in other variables were found between environments (all *ps *> 0.05, Figure 3). There were no significant differences in the number of responses (*p *= 0.2) or accuracy of texting (*p *= 0.3) responses between the indoor and outdoor environments.

**Table 2:** Median (interquartile range) values are presented for the dependent variables across indoor and outdoor.

	Indoor TUG_S_	Outdoor TUG_S_	*p*-value, effect size	Indoor TUG_TEXT_	Outdoor TUG_TEXT_	*p*-value, effect size
Gait Speed (m/sec)	2.1 (1.5–2.9)	2.2 (1.7–3.0)	*p *= 0.2, ES = 0.3	1.7 (1.1–2.1)	1.6 (1.2–1.9)	*p *= 0.2, ES = 0.2
Gait Speed DTC (%)				−30.3 (−40.3–−12.7)	−37.0 (−45.6–−12.4)	*p *= 0.1, ES = 0.3
Step count (step)	11.0 (10–12.7)	11.0 (10.0–12.7)	*p *= 0.1, ES = 0.3	14.0 (13.0–15.7)	14.0 (13.0–16.0)	*p *= 0.74, ES = 0.1
Step count DTC (%)				−24.7 (−36.4–−17.0)	−29.3 (−39.1–−18.6)	*p *= 0.2, ES = 0.3
Sit-to-stand Time (sec)	1.2 (1.0–1.4)	1.3 (0.9–1.4)	*p *= 0.8, ES = 0.05	1.2 (1.1–1.4)	1.2 (1.2–1.4)	*p *= 0.94, ES = 0.01
Sit-to-stand DTC (%)				−2.7 (−19.2–13.7)	0.8 (−10.9–13.7)	*p *= 0.2, ES = 0.3
Walk time (sec)	1.4 (1.0–1.9)	1.2 (0.9–1.6)	*p *= 0.01, ES = 0.6	1.7 (1.3–2.6)	1.8 (1.5–2.5)	*p *= 0.6, ES = 0.1
Walk Time DTC (%)				−43.8 (−67.7–−14.7)	−64.0 (−89.2–−44.0)	*p *= 0.008, ES = 0.7
Turn Time (sec)	1.1 (0.9–1.4)	1.1 (0.9–1.5)	*p *= 0.9, ES = 0.008	1.5 (1.2–1.9)	1.5 (1.3–1.8)	*p *= 0.09, ES = 0.4
Turn Time DTC (%)				−23.9 (−58.8–−7.8)	−24.9 (−42.8–−2.3)	*p *= 0.057, ES = 0.4
Stand-to-sit Time (sec)	0.8 (0.7–1.0)	0.8 (0.6–1.0)	*p *= 0.4, ES = 0.2	0.8 (0.7–1.1)	0.8 (0.6–1.1)	*p *= 0.4, ES = 0.2
Stand-to-sit Time DTC (%)				−3.3 (−12.8–19.9)	−10.2 (−21.5–17.4)	*p *= 0.2, ES = 0.3
Number of Responses in Texting Task				6.0 (5.0–7.75)	7.0 (5.25–8.75)	*p *= 0.2, ES = 0.3
Accuracy (%)	—			73.3 (51.8–83.0)	67.5 (44.6–87.1)	*p *= 0.35, ES = 0.2

DTC, dual-tasking cost; TUGS, single-task TUG; TUGVF, TUG with verbal fluency task; TUGTEXT, TUG with texting task.

## Discussion

The effect of texting in an outdoor environment has not yet been quantified using different *dynamic tasks* such as turning, sitting-to-standing, or standing-to-sitting. We considered two factors related to motor behavior in the present investigation, the task (texting) and environment. Specifically, we examined whether texting affects dynamic task performance in outdoor and indoor environments equally. The study population consisted of healthy adults to minimize inter-individual variability as dual-tasking is age-dependent (5, 18). The results supported the hypothesis that the DTC of walking time was higher (i.e., poorer performance) outdoors compared to indoors while texting.

This is the first study to examine the effect of the environment using four different dynamic tasks: sit-to-stand, walk, turn, and stand-to-sit. Our results indicate that attentional resources vary according to the type of dynamic task (e.g., walking vs. turning or other tasks) and the environment. We found that more attentional resources were required for walking in an outdoor environment compared to sit-to-stand, turning, and stand-to-sit, relative to indoors. This reflects the inherent challenge of the walking task, especially when anticipation and transitions to other dynamic tasks are needed in the outdoor environment. When walking outdoors, the participants encountered higher demands for updating their motor strategies based on any unpredictable events and distractions, e.g., traffic noise, pedestrians, or moving cars. These distractors were not present in the predictable laboratory environment, requiring far less attention. In line with this finding, a previous study reported that older adults (aged 65–80 years) showed greater gait variability in an outdoor environment than younger adults (5). Although not quite significant, a trend in our data suggested that turning during outdoor activities requires higher attentional demands than indoors. Previous dual-tasking TUG studies reported greater dual-tasking decrements on walking and turning tasks compared to sit-to-stand or stand-to-sit (18, 33). The walking task in the TUG test takes the longest time, followed by turning, suggesting that the DTC is time-sensitive (18). This implies that a longer task may provide additional opportunities for individuals to be affected by the distractions of outdoor environments, making more errors in dual-tasking conditions. It should be noted that previous research showed fair to excellent test-retest reliability for time and gait speed when completing dual-task TUG tests, however; poor to good test-rest reliability for DCTs were reported (34, 35). This may occur due to an increase in systematic errors when the difference between the single and dual-task conditions is calculated to measure the DTCs (36).

Interestingly, our results disagree with previous studies that reported no environmental effect on younger adults' performance (5–7). This may be due to two reasons. First, the walking task in our protocol required acceleration and deceleration and is different from the straight-line walking tasks used in previous research (5–7). Texting creates interference to the visual system, and the individuals can ignore up to half of the visual cues within the environment (37). The TUG test imposes greater challenges because it requires more planning and dependence on the visual system to transitions between subtasks and avoid hitting the cone and chair. Second, we chose to explore the effect of the environment on a cohort with a mean age of 38.3 years and asked the participants to use their personal mobiles to complete the texting task. Previous studies used a lower average age of the sample and one mobile device across all the participants to complete the texting task (5–7). Future research is required to determine the effect of outdoor environments on dynamic tasks in older adults, which may lead to the development of better rehabilitation protocols for those at risk of falls.

Consistent with previous research (5–7), the accuracies of texting did not vary between environments. This suggests that the attentional focus of young and middle-aged adults shifted more toward completing the TUG rather than texting, as evidenced by a poorer performance of walking while texting outdoors relative to indoors. One advantage of the texting task is its ecological validity, which can be viewed as disadvantageous as it creates variability within the individuals, e.g., the length of written sentences. Thus, we cannot rule out the possibility that the accuracy of texting was comparable in both environments because the participants strategically adapted to the complexity of walking while texting by typing shorter and easier sentences outdoors.

We recognize that the results of this study should be considered in the context of its shortcomings. First, we examined a small sample of highly educated individuals within a very large age range (25–62 years old). Additionally, 65% of the participants had prior knowledge of the TUG, which may have affected their motor performance. Although this study was not powered to assess the aging effect, age difference should be explored thoroughly in future studies. This caveat limits the generalizability of the findings. However, if dual tasking in outdoor environments can impact active, educated individuals, then more significant interference may be expected in frail older adults. Second, while navigating a parking lot is certainly a real-world activity, our “outdoor” environment did not adequately represent other challenging environments, such as crossing a busy street or shopping in a grocery store, and the difference in the baseline metrics could create the difference in dual tasking costs in walking. Further, we only assessed one trial per condition in this study, and the questions we used in texting tasks might create inequivalent cognitive loads, i.e., in indoor environment, the questions asked about activities in the past, and in outdoor environment, the question asked about activities in future. In the future, we plan to use cognitive tasks with equivalent cognitive load and assess more than one trial per condition and perform the outdoor portion of this experiment on a sidewalk next to a busy road to better simulate a real-world scenario, as it would include more noise, distractions, and pedestrian traffic. Lastly, despite using a questionnaire to document texting skill levels, we did not measure the single-task performance for texting, which would have improved the study quality.

To conclude, in this study, we examined the effect of texting on four functional activities in indoor (laboratory) and outdoor (real) environments. We found that texting in an outdoor environment can negatively impact walking time when integrated with other dynamic tasks. However, our results revealed that there was no significant difference in the dual-task costs when completing sitting-to-standing, turning, and standing-to-sitting activities, suggesting comparable cognitive load. Texting while walking increases the accidental risks and compromises pedestrian safety (3, 4). Since we cannot eliminate texting due to the common use of technologies, our emphasis should be shifted toward educating pedestrians about the attentional demands of texting in dynamic, busy environments.

## Data Availability

The raw data supporting the conclusions of this article will be made available by the authors, without undue reservation.
